# Bilateral Traumatic Anterior Dislocation of the Hip with an Unstable Lumbar Burst Fracture

**DOI:** 10.4055/cios.2009.1.2.114

**Published:** 2009-05-27

**Authors:** Kook Jin Chung, Sang Wha Eom, Kyu Cheol Noh, Hong Kyun Kim, Ji Hyo Hwang, Hoi Soo Yoon, Jung Han Yoo

**Affiliations:** Department of Orthopaedic Surgery, Kangnam Sacred Heart Hospital, College of Medicine, Hallym University, Seoul, Korea.; * Department of Radiology, Hallym University Sacred Heart Hospital, College of Medicine, Hallym University, Seoul, Korea.

**Keywords:** Hip dislocation, Anterior, Traumatic, Bilateral

## Abstract

Traumatic anterior dislocation of the hip is rare. Bilateral traumatic anterior dislocation is an even rarer injury; indeed, only 5 cases have been reported in the English literature. We describe a case of a bilateral traumatic anterior dislocation of the hip and a concomitant unstable lumbar burst fracture following a mechanism of injury distinctly different from other reports.

Hip dislocations are orthopaedic emergencies because of the possible complications. The usual direction of a hip dislocation is posterior and the rate of posterior dislocations is 90%.1) Bilateral anterior dislocation of the hip is an extremely rare injury. In fact, there have been no reports of a bilateral traumatic anterior dislocation of the hip and a concomitant unstable lumbar burst fracture. We therefore describe a case involving a bilateral traumatic anterior dislocation of the hip and concomitant unstable lumbar burst fracture following a mechanism of injury which was distinctly diff erent from other reports.

## CASE REPORT

**Fig. 1:**
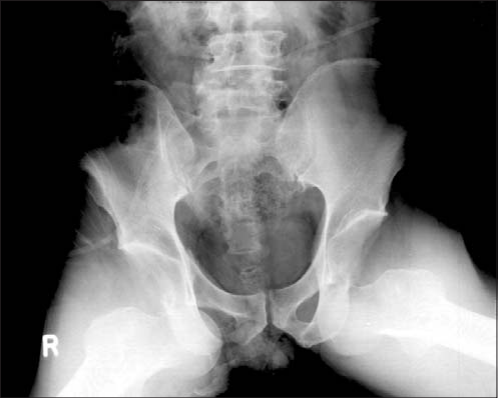
Radiograph showed bilateral anterior dislocation of both hip joints and disruption of the axial alignment at L4/5.

**Fig. 2:**
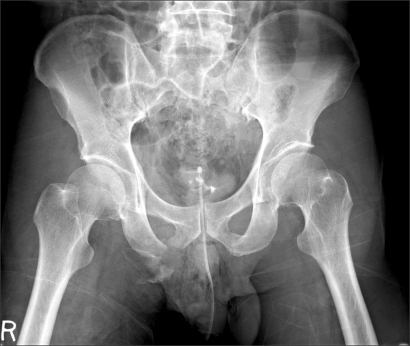
Radiograph showed concentric reduction of both hips

**Fig. 3:**
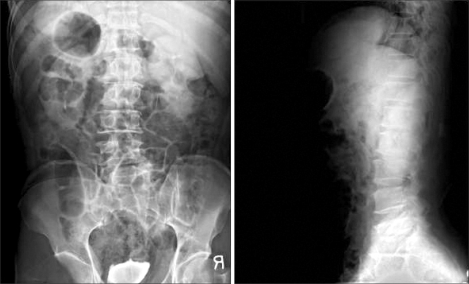
Radiograph showed axial malalignment and decreased vertebral body height of L5.

**Fig. 4:**
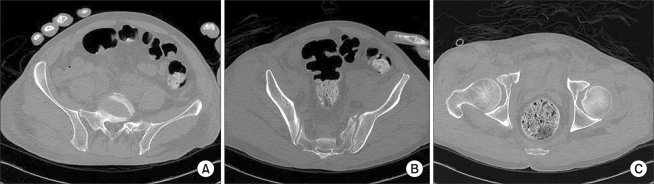
Pelvis computed tomography showed extra-articular fracture of the right acetabulum (A), sacral alar (B), and anterior portion of the sacroiliac joint on the left (C).

**Fig. 5:**
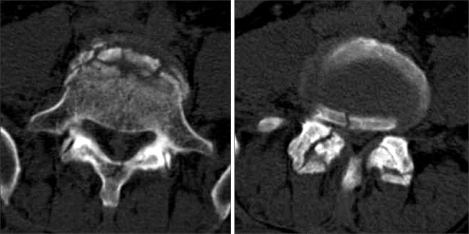
Computed tomography showed an unstable burst fracture of L5. Fracture of the L5 body with a greenstick lamina fracture (A) and bilateral inferior articular process fracture of L4 (B).

**Fig. 6:**
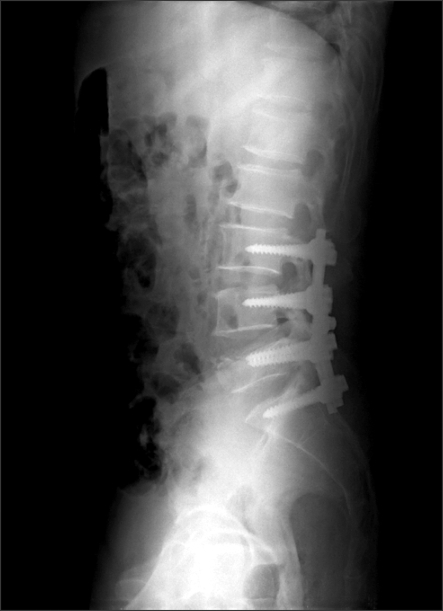
Postoperative radiographs showed posterior segmental fixation from L3 to S1.

A 60-year-old man who was working in a squatting position at a construction site was struck by a 600 lb H-beam falling from 2 m over his lower back and buttocks area. When he arrived at our hospital 3 hours after the injury, he already had been diagnosed with a bilateral anterior dislocation of the hip (Fig. 1) and a spine fracture. He was transferred to our hospital from a private clinic with the right hip reduced, but the left hip remained unreduced. On arrival at our hospital, the patient had severe lower back pain and bilateral hip pain, but the vital signs were stable and there were no neurologic deficits. Roentgenographic studies were obtained in the department of emergency medicine and the plain pelvis film showed that the right hip was reduced, but the left femoral head was displaced over the obturator foramen; a right superior and inferior ramus fracture, and a right acetabulum fracture were noted (Fig. 2). The lumbar spine film showed disruption of the axial alignment and decreased vertebral body height of L5 (Fig. 3). A pelvic computed tomography (CT) demonstrated an extra-articular fracture of the right acetabulum, sacral alar, and the anterior portion of the sacroiliac joint on the left. On lumbar spine CT, there was canal encroachment by the fractured body of L5 (Fig. 4), a greenstick laminar fracture, bilateral inferior articular process fractures of L4, and a left sacral alar fracture (Fig. 5). Closed reduction was achieved under general anesthesia by longitudinal traction on the left leg while an assistant applied lateral traction on the upper thigh while simultaneously pushing the femoral head toward the acetabulum. After reduction, posterior segmental fixation was done from L3 to S1 on the unstable lumbar burst fracture (Fig. 6). Bilateral Buck's traction was applied for 6 weeks, followed by non-weight bearing for 6 weeks. Three months after the injury, he gained full range of motion on both hip joints and there were no complications at the 2 year follow-up.

## DISCUSSION

Dislocation of the hip joint represents 2% to 5% of all traumatic dislocations.2) Bilateral hip dislocation rarely occurs; indeed, there have only been five cases reported in the English literature.^3^-7)

Motor vehicle accidents were the cause of bilateral traumatic anterior dislocations of the hip in all reported cases.^4^-7) Unlike posterior dislocations of the hip, several authors have reported that an obturator-type of anterior dislocation occurs when flexion exists in association with external rotation and abduction.^4^-8) Our patient was struck over the lumbosacral and buttocks area by a heavy object weighing 600 lb. We thought that this direct blow made the spinal column unstable and the resultant direct pressure by the heavy object forced both hip joints to be dislocated anteriorly, while both lower extremities were fixed with both hips flexed, abducted, and externally rotated.

Except for an unreduced bilateral anterior dislocation,3) all cases of bilateral anterior dislocation were reduced by a closed method. There was a case of asymmetric bilateral dislocation in which open reduction was needed because the femoral head was button-holed through the anterior capsule.9)

A hip joint dislocation is an orthopaedic emergency. Delay in the reduction of a dislocated hip joint increases the incidence of avascular necrosis, which develops in 26% of hip dislocations.2) When we try to reduce anterior dislocations of the hip, we have to bear in mind that prompt and gentle reduction under adequate intravenous or general anesthesia and a stepwise procedure with traction in line with the femur, gentle flexion of the hip joint with continued traction, and gentle internal rotation and abduction, is key to avoiding possible complications.

This case report is without precedent and illustrates that a rare bilateral anterior dislocation of the hip can occur following unusual mechanisms of injury.
